# Spatial variation of skilled birth attendance and associated factors among reproductive age women in Ethiopia, 2019; a spatial and multilevel analysis

**DOI:** 10.3389/fgwh.2024.1082670

**Published:** 2024-08-12

**Authors:** Fantu Mamo Aragaw, Gela Atlie, Amensisa Hailu Tesfaye, Daniel Gashaneh Belay

**Affiliations:** ^1^ Department of Epidemiology and Biostatistics, Institute of Public Health, College of Medicine and Health Sciences, University of Gondar, Gondar, Ethiopia; ^2^ Department of Internal Medicine, College of Medicine and Health Sciences, Madda Walabu University, Goba, Oromia, Ethiopia; ^3^ Department of Environmental and Occupational Health and Safety, Institute of Public Health, College of Medicine and Health Sciences, University of Gondar, Gondar, Ethiopia; ^4^ Department of Human Anatomy, College of Medicine and Health Sciences, University of Gondar, Gondar, Ethiopia

**Keywords:** skilled birth attendance, women, spatial variation, multilevel analysis, Ethiopia

## Abstract

**Background:**

The majority of maternal deaths were associated with a lack of access to skilled birth attendance. Because childbirth accounts for most maternal deaths, skilled birth attendance is crucial for reducing maternal mortality. The use of skilled birth attendance in Ethiopia is low, and it is crucial to identify factors that determine the use of skilled birth attendance. Hence, this study aimed to assess the spatial distribution, wealth-related inequality, and determinants for skilled birth attendance in Ethiopia.

**Methods:**

Secondary data analysis was done with a total weighted sample of 5,251 reproductive-aged women using the 2019 mini EDHS. The concentration index and graph were used to assess wealth-related inequalities. Spatial analysis was done to identify the spatial distribution and multilevel logistic regression analysis was used to identify predictors of skilled birth attendance in Ethiopia. Analysis was done using STATA version 14, ArcGIS, and SaTscan software.

**Results:**

The prevalence of skilled birth attendance was 50.04% (95% CI: 48.69%, 51.40%) in Ethiopia. Old age, being married, being educated, having television and radio, having ANC visits, being multiparous, having large household sizes, having a rich wealth index, living in rural residence, and living in a high level of community poverty and women's education were significant predictors of skilled birth attendance. Skilled birth attendance was disproportionately concentrated in rich households [C = 0.482; 95% CI: 0.436, 0.528]. High prevalence of unskilled birth attendance was found in Somalia, SNNP, Afar, and southern parts of the Amhara regions. Primary clusters of unskilled birth attendance Somalia and some parts of Oromia region of Ethiopia.

**Conclusion:**

Half of the women in Ethiopia did not utilize skilled birth attendants with significant spatial clustering. Age, marital status, educational status, ANC Visit, having television and radio, parity, household size, wealth index, residence, community level poverty, and community level of women's education were significant predictors of skilled birth attendance. Skilled birth attendance was unevenly concentrated in rich households. The regions of Somalia, SNNP, Afar, and southern Amhara were identified as having a high prevalence of using unskilled birth attendance. Public health interventions should target those women at high risk of using unskilled birth attendants.

## Introduction

Maternal mortality remains a major challenge for global health systems particularly in developing countries (1). One of the primary causes of high maternal and neonatal mortality rates worldwide is a lack of access to healthcare services during pregnancy and delivery (2–4). The majority of maternal deaths are caused by obstetric complications, particularly in low-income countries (5). The majority of maternal deaths occur in Sub-Saharan Africa, with the majority of these deaths being associated with a lack of access to antenatal care and skilled birth attendance (6).

Skilled birth attendance (SBA) is defined as the process by which a skilled health professional such as a doctor, nurse, or midwife provides adequate care to a woman during labor, delivery, and the early postpartum period (7). Because the majority of maternal deaths and obstetric complications occur during delivery, SBA remains the most important intervention in reducing maternal mortality and complications (8). Skilled birth attendant-assisted delivery care has been identified as a protective mechanism for saving maternal and newborn lives (9–11). According to studies, skilled birth attendance can considerably reduce obstetric complications such as stillbirths (12, 13).

Skilled birth attendance is a well-known method of reducing maternal and perinatal mortality and morbidity (14–17). A skilled delivery service provides a safe environment for both mothers and newborns, lowering the likelihood of complications (18). As a result, skilled birth attendance is critical and the best strategy for reducing maternal and perinatal deaths (19–21). Variables such as educational status (22–25), wealth index (23, 26–28), residence (28–30), ANC visit (22, 31–35), media exposure (28, 32, 36) were identified as significant predictors of skilled birth attendance.

Studies indicated that maternal mortality is typically low when a greater proportion of deliveries are attended by skilled birth attendants (37–39). Even though the Ethiopian government implemented several interventions, such as training skilled health providers and making most maternal health services free of charge, skilled birth attendance remains a major challenge in Ethiopia. Therefore, this study aimed to assess the spatial distribution; wealth-related inequality, and determinants for skilled birth attendance among reproductive-age women in Ethiopia using nationally representative data. The study's findings will support policymakers in the implementation of measures that will enhance the utilization of skilled delivery care in Ethiopia.

## Methods

### Study design, study period, and study setting

A community-based cross-sectional study was conducted in Ethiopia using the 2019 mini-EDHS data conducted from March 21, 2019, to June 28, 2019. The study was conducted in Ethiopia is located in the horn of Africa (3^o^–14^o^ N and 33^o^–48^°^E). Ethiopia is the second-most populous country in Africa with nine regional states and two city administrations (Addis Ababa and Dire Dawa). Administratively, regions are divided into zones, zones into woredas, and woredas into the lowest administrative unit known as kebeles.

### Data source

The data for this study were retrieved from EDHS data of 2019, which was the second EMDHS and the fifth DHS implemented in Ethiopia. Data were obtained from the DHS website: www.dhsprogram.com by justifying the reason for requesting the data and after obtaining an approval letter from the DHS. For this study, we used the individual record data set.

### Sampling procedures and populations

The source population was all women of reproductive age who gave birth in Ethiopia within five years before the survey, while the sample population was all women of reproductive age who gave birth in the selected enumeration areas (EAs) within five years before the survey. Samples were selected using a stratified, two-stage cluster design, using EAs as primary sampling units and households as secondary sampling units.

Each region was divided into urban and rural areas, resulting in 21 different sampling strata. Firstly, a total of 305 EAs (93 in urban, 212 in rural) were chosen independently with a probability proportional to each EA. Second, from the newly formed household listing, a fixed number of 30 households/clusters were selected with an equal probability of systematic selection. The detailed sampling procedures are available on the measure DHS website in the 2019 EMDHS report (https://www.dhsprogram.com). A total weighted sample of 5,251 reproductive-age women who gave birth within five years of the survey was included.

### Variables of the study

The outcome of this study was delivery by a skilled birth attendant. Skilled birth attendance refers to births delivered with the assistance of doctors, nurses/midwives, health officers, and health extension workers (40). If the woman received the assistance of skilled personnel (i.e., doctor, midwives/nurse, public health officer, and health extension) during my most recent childbirth” they coded as “yes”, otherwise “no”.

Individual and community-level independent variables were considered in this study. Maternal age, marital status, maternal educational level, household size, household head, television, radio, ANC visit, household wealth, and parity were some of the individual-level factors included in this study.

Community-level factors residence, community-level poverty, community level of women's education, and region were included. In this study region was categorized into three categories; larger central [Tigray, Amhara, Oromia, and Sothern Nations Nationalities and Peoples Region], small peripherals [Afar, Somali, Benishangul Gumuz, and Gambela], and metropolis [Harari, Dire Dawa, and Addis Ababa] based on their geopolitical features, which is consistent with a prior Ethiopian study (41–43).

Community-level poverty was the proportion of women in the community who live in the lower (poor) and higher (rich) quintiles of the wealth index are classified as high (proportion of women greater than the median national value) or low (proportion of women less than the median national value) (44). The community level of women's education was the proportion of women in the community with at least a primary level of education, categorized as high (proportion of women greater than median national value) whereas low (proportion of women below-median national value) (44).

### Data management and analysis

The data was cleaned to ensure consistency with the EMDHS 2019 report. Data were weighted before statistical analysis using sampling weight (v005), primary sampling unit (v021), and strata (v023) to restore the survey's representativeness and obtain valid statistical estimates. Descriptive and summary statistics were done using STATA version 14 software.

### Spatial analysis

#### Spatial distribution and autocorrelation

The global spatial autocorrelation (Global Moran's I) was used to determine whether skilled birth attendance patterns were dispersed, clustered, or randomly distributed in the study area (45). Moran's I value close to −1 indicate the spatial distribution of unskilled birth attendance is dispersed, whereas Moran's I value close to +1 indicate unskilled birth attendance is clustered (46). The Moran I value close to 0 means the spatial distribution of unskilled birth attendance is random. A statistically significant Moran's I (*p* < 0.05) indicates the spatial clustering of unskilled birth attendance.

The maximum peak distance where unskilled birth attendance clustering is more pronounced was determined using incremental spatial autocorrelation. The maximum peak distance is the distance at which the spatial autocorrelation is maximum, and it was used as a distance band for hot-spot analysis. A total of ten distance bands were identified, with a beginning distance of 155,205 m, the first peak at 299,451.07 m, and peak clustering at 299,451.07 m.

#### Hot spot and cold spot analysis (Getis-Ord Gi* statistics)

Gettis-Ord Gi* statistics were computed for each area to determine how spatial autocorrelation varies across the study location. Z-score was calculated to ensure the statistical significance of clustering, A positive z-score >1.96 with significant *p*-values indicates a hotspot, whereas a negative z-score >1.96 with significant *p*-values indicates a cold spot (47, 48). The hot spot areas indicated areas with a high proportion of unskilled birth attendance and the cold spot ones indicated that there was a low proportion of unskilled birth attendance.

#### Spatial interpolation

In this study, we used a geostatistical ordinary Kriging spatial interpolation technique to predict values for areas where data points were not taken. Interpolation is based on the assumption that spatially distributed objects are spatially correlated; in other words, things that are close together tend to have similar characteristics (49, 50). Based on sampled clusters, the spatial interpolation technique is used to predict unskilled birth attendance for unsampled areas.

#### Spatial scan statistical analysis

Using Kuldorff's SaTScan version 9.6 statistical software, a Bernoulli-based model was used to identify statistically significant spatial clusters of unskilled birth attendance. To fit the Bernoulli model, women who had unskilled birth attendance were considered cases, while those who had skilled birth attendance were considered controls. The default maximum spatial cluster size of <50% of the population was used as an upper limit, allowing for the detection of both small and large clusters while ignoring clusters that contained more than the maximum limit. A likelihood ratio test statistic and the *p*-value were used for each potential cluster to determine whether the number of observed unskilled birth attendants within the potential cluster was significantly higher than expected.

#### Parameter estimation method

The random effects are measures of variation in skilled birth attendance across communities or clusters, were expressed in terms of the Intra-Class Correlation (ICC), the median odds ratio (MOR), and the proportional change in variance (PCV) (51–53). MOR is defined as the central value of the odds ratio between the greatest and lowest risk regions when two clusters are selected at random. The PCV explains the variability in skilled birth attendance among reproductive-aged women. The ICC shows the differences between clusters in skilled birth attendance among reproductive-aged women (54, 55).

#### Multilevel analysis

We used a multilevel logistic regression analysis to account for the heterogeneity between clusters in the EDHS data. Variables with *p*-value <0.2 in the bi-variable analysis for both individual and community-level factors were fitted in the multivariable model. Adjusted Odds Ratio (AOR) with a 95% Confidence Interval (CI) in the multivariable model was used to declare statistically significant associations with our outcome variable. Multi-collinearity was checked using the variance inflation factor (VIF) by conducting a pseudo-linear regression analysis, with a cut-off value of <5.

#### Model building

Four models have fitted: the null model (models without independent variables), model I (models with individual-level variables), model II (models include community-level variables), and model III (models with both individual and community-level variables). Deviance was used to assess model fitness since these models were nested. The variance inflation factor (VIF) was used to detect multicollinearity.

#### Concentration index and curve

The concentration index measures the magnitude and direction of socioeconomic inequality in a health variable. A negative sign indicates that the poor have a higher concentration of skilled birth attendance; whereas a positive sign indicates that the wealthy have a higher concentration of skilled birth attendance.

#### Ethical consideration

All methods were carried out following relevant guidelines of the Demographic and Health Surveys (DHS) program. Informed consent was waived from the International Review Board of Demographic and Health Surveys (DHS) program data archivists after the consent paper was submitted to the DHS Program, a letter of permission to download the dataset for this study. The dataset was not shared or passed on to other bodies and has maintained its confidentiality.

## Results

A total weighted sample of 5,251 women was included in the study. The prevalence of skilled birth attendance among women was 50.04% (95% CI: 48.69%, 51.40%) in Ethiopia. More than half, 2,829 (53.88%) were between the ages of 25 and 34 years. More than half of the participants 2,801 (53.36%) of the women had no formal education**.** Women who had secondary and higher education were more likely to have skilled birth attendance 512(86.74%) than those who had no formal education 993(35.43%). Women with a rich wealth index of 1,414 (74.99%) were more likely to have skilled birth attendance than those who had a poor wealth index 745 (31.49%). Around three fourth of the participants 3,932 (74.89%) lived in a rural area. Women who live in rural areas were less likely to have skilled birth attendance, 1,679 (42.72%) than those who live in urban areas 948 (71.93%) (Table 1).

**Table 1:** Characteristics of the study population with skilled birth attendance among reproductive-age women, 2019 mini EDHS.

Variables	Categories	Skilled birth attendance		Total weighted frequency (%)
		Yes (%)	No (%)	
		*n* = 2,628 (50.05)	*n* = 2,623 (49.95)	
Age of women	15–24	706 (57.83)	514 (42.17)	1,221 (23.26)
	25–34	1,385 (48.97)	1,443 (51.03)	2,829 (53.88)
	35–49	536 (44.68)	664 (55.32)	1,200 (22.86)
Marital status	Married	2,466 (49.97)	2,469 (50.03)	4,936 (94.01)
	Not married	119 (51.28)	153 (48.72)	314 (5.99)
Women education status	No education	993 (35.43)	1,809 (64.57)	2,801 (53.36)
	Primary	1,122 (60.42)	735 (39.58)	1,857 (35.38)
	Secondary and higher	512 (86.74)	78 (13.26)	591 (11.26)
Parity	Primi	628 (75.00)	209 (25.00)	838 (15.96)
	Multi	1,999 (45.31)	2,413 (35.55)	4,412 (84.04)
Household size	Less than six	1,482 (62.11)	904 (37.89)	2,387 (45.47)
	Greater than or equal to six	1,145 (39.99)	1,718 (60.01)	2,863 (54.53)
Sex of household head	Male	2,257 (49.70)	2,285 (50.30)	4,542 (86.51)
	Female	370 (52.30)	337 (47.70)	708 (13.49)
Have radio	Yes	842 (63.03)	494 (36.97)	1,337 (25.47)
	No	1,785 (45.61)	2,128 (54.39)	3,913 (74.53)
Have television	Yes	682 (88.42)	89 (11.58)	772 (14.71)
	No	1,945 (43.43)	2,533 (56.57)	4,478 (85.29)
ANC visit	Yes	2,491 (57.84)	1,815 (42.16)	943 (17.97)
	No	136 (14.50)	806 (85.50)	4,307 (82.03)
Wealth index	Poor	745 (31.49)	1,622 (68.51)	2,368 (45.10)
	Middle	467 (46.94)	528 (53.06)	996 (18.97)
	Rich	1,414 (74.99)	471 (25.01)	1,886 (35.92)
Community level variables				
Residence	Urban	948 (71.93)	370 (28.07)	1,318 (25.11)
	Rural	1,679 (42.72)	2,252 (57.28)	3,932 (74.89)
Community level poverty	Low	2,008 (63.38)	1,160 (36.62)	3,169 (60.35)
	High	619 (29.75)	1,462 (70.25)	2,081 (39.65)
Community level of women's education	Low	816 (34.08)	1,578 (65.92)	2,394 (45.60)
	High	1,811 (63.43)	1,044 (36.57)	2,856 (54.40)
Region	Larger central	2,275 (50.33)	2,245 (49.67)	4,520 (86.09)
	Small peripherals	175 (32.86)	358 (67.14)	534 (10.18)
	Metropolis	177 (90.34)	18 (9.66)	196 (3.74)

### Random effect and model comparison

In the null model, the ICC indicated that 57% of the total variability for skilled birth attendance was due to differences between clusters while the remaining unexplained 43% of the total variability of skilled birth attendance was attributable to individual differences. Furthermore, the PCV value in the final model indicates that about 59.1% of the variations in skilled birth attendance among study subjects were attributed to both individual and community-level factors. Model comparison and fitness was used. The model with the lowest deviance was the best-fitted model, which was model three (4,631). Multi-collinearity was not found since all variables had a VIF of less than five (Table 2).

**Table 2:** Model fitness and random effect measures.

Measure of variation	Null model	Model I	Model II	Model III
VA	4.39	1.93	2.17	1.76
ICC	0.57	0.37	0.39	0.34
MOR	5.40	3.56	3.77	3.42
PCV (%)	–	49.9%	50.5%	59.1%
Model fitness and comparison				
Deviance	5,334	4,696	5,153	4,631
Mean VIF	–	1.46	1.49	1.71
ICC, inter cluster corrolation cofficent; MOR, median odds ratio; PCV, proportional change in variance; VIF, variance inflation factor				

### Wealth-related inequality of skilled birth attendance

#### Concentration index and curve

**Figure 1:**
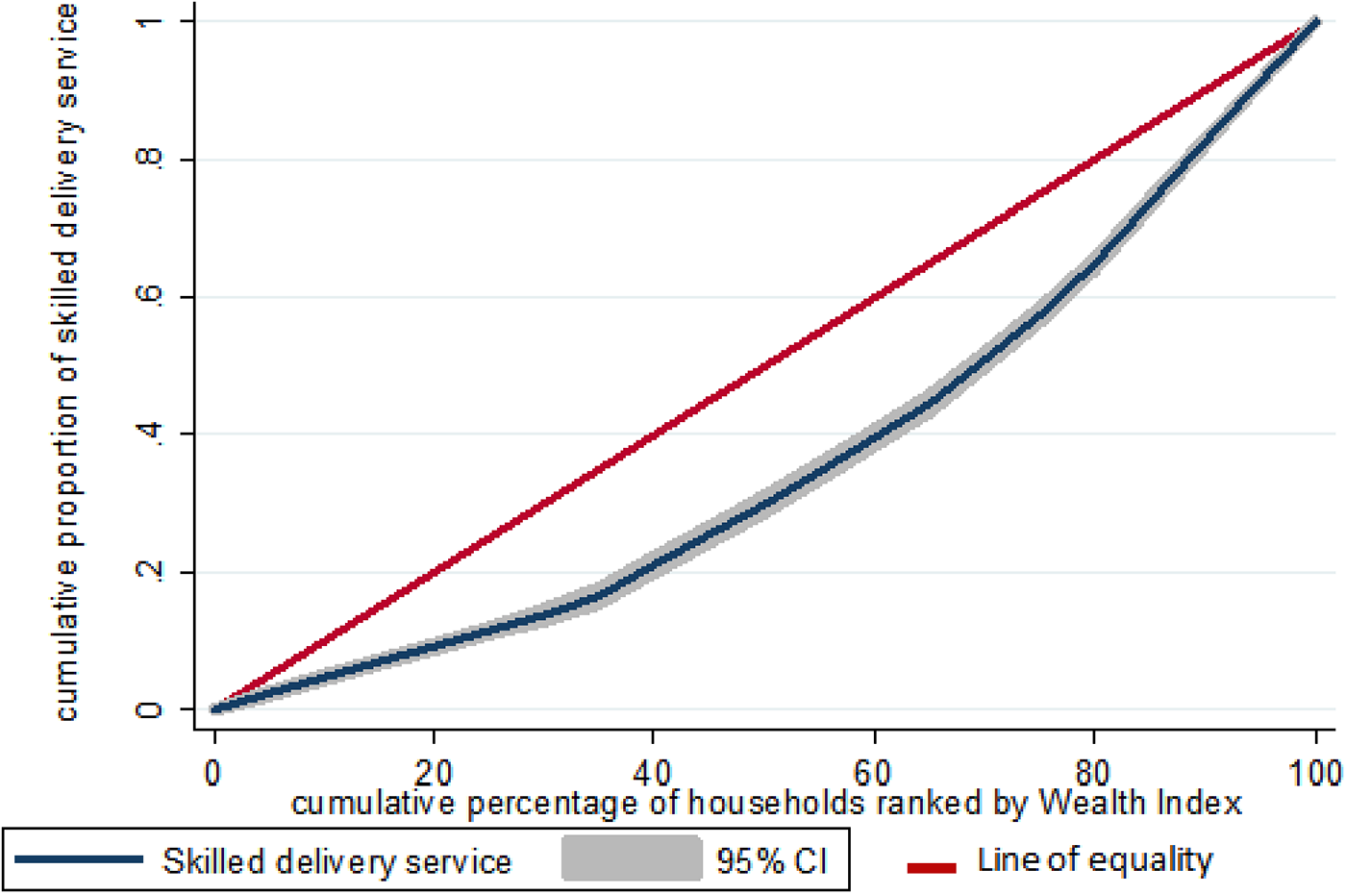
Wealth-related inequality of skilled birth attendance among reproductive-age women in Ethiopia, 2019 mini EDHS.

The value of a negative sign in the concentration index indicates a more concentration of skilled birth attendance among the poor, whereas a positive value indicates concentration among the rich. In this study, the overall wag staff normalized concentration index (C) analyses of the wealth-related inequality of skilled birth attendance showed the pro-rich distribution of skilled birth attendance with [C = 0.482; 95% CI: 0.436, 0.528]. This shows that skilled delivery care utilization among women was disproportionately concentrated among the richer groups (pro-rich). Similarly, the concentration curve in the figures showed that the concentration graph of skilled birth attendance was below the line of equality which indicated that the distribution of skilled birth attendance was concentrated in rich households (pro-rich distribution) (Figure 1).

#### Spatial analysis results

##### Spatial and incremental autocorrelation analysis

**Figure 2:**
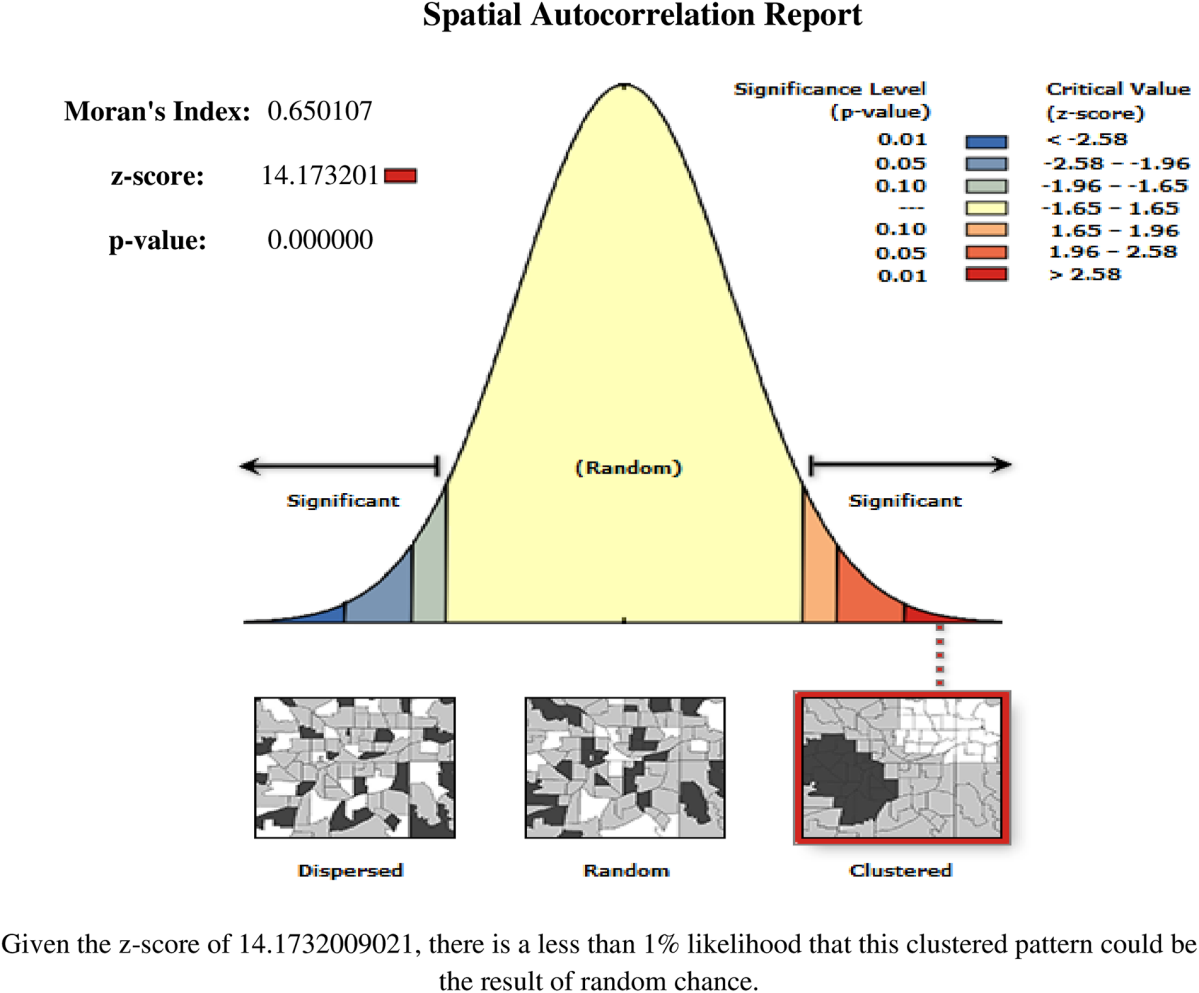
Spatial autocorrelation analysis of non-skilled birth attendance among reproductive-age women in Ethiopia, 2019 mini EDHS.

Based on the 2019 mini-EDHS, the spatial patterns of unskilled birth attendance in Ethiopia revealed a significant spatial heterogeneity across the country over regions, which was found to be non-random with Global Moran's I value of 0.65 with (*p* < 0.00001) (Figure 2). The incremental autocorrelation result revealed statistically significant z-scores at a peak distance of 299.451 km 4.273 (distances; Z-score) for skilled birth attendance.

##### Spatial distribution of skilled birth attendance

**Figure 3:**
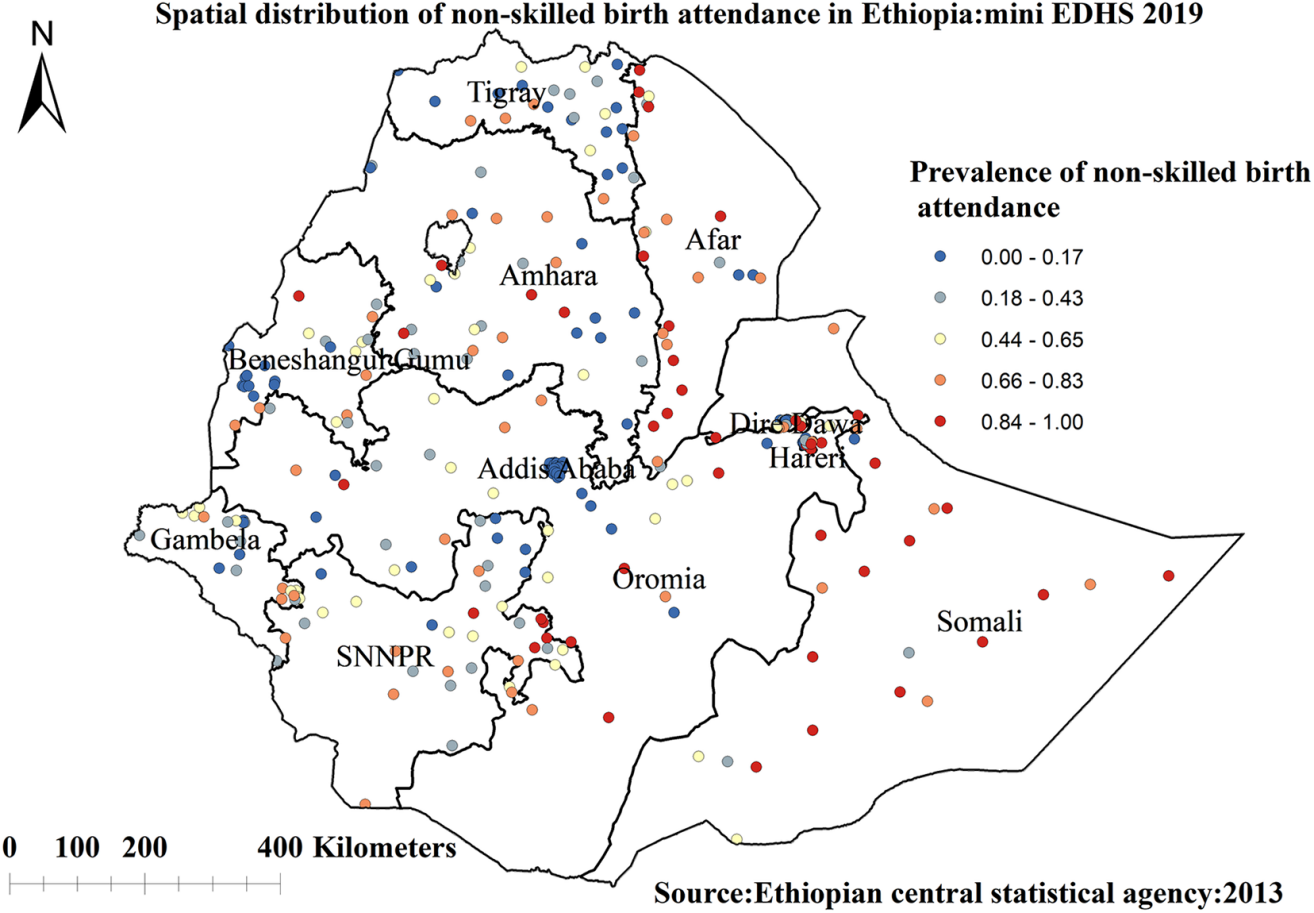
Spatial distribution of non-skilled birth attendance among reproductive-age women in Ethiopia, 2019 mini EDHS.

For the spatial analysis of skilled birth attendance, a total of 305 clusters were considered. Each point on the map represents one enumeration area with a proportion of unskilled birth attendance in each cluster. The red color represents areas with a high proportion of unskilled birth attendance, while the blue color represents EAs with a lower proportion of skilled birth attendance (Figure 3).

##### Hotspot and cold spot analysis

**Figure 4:**
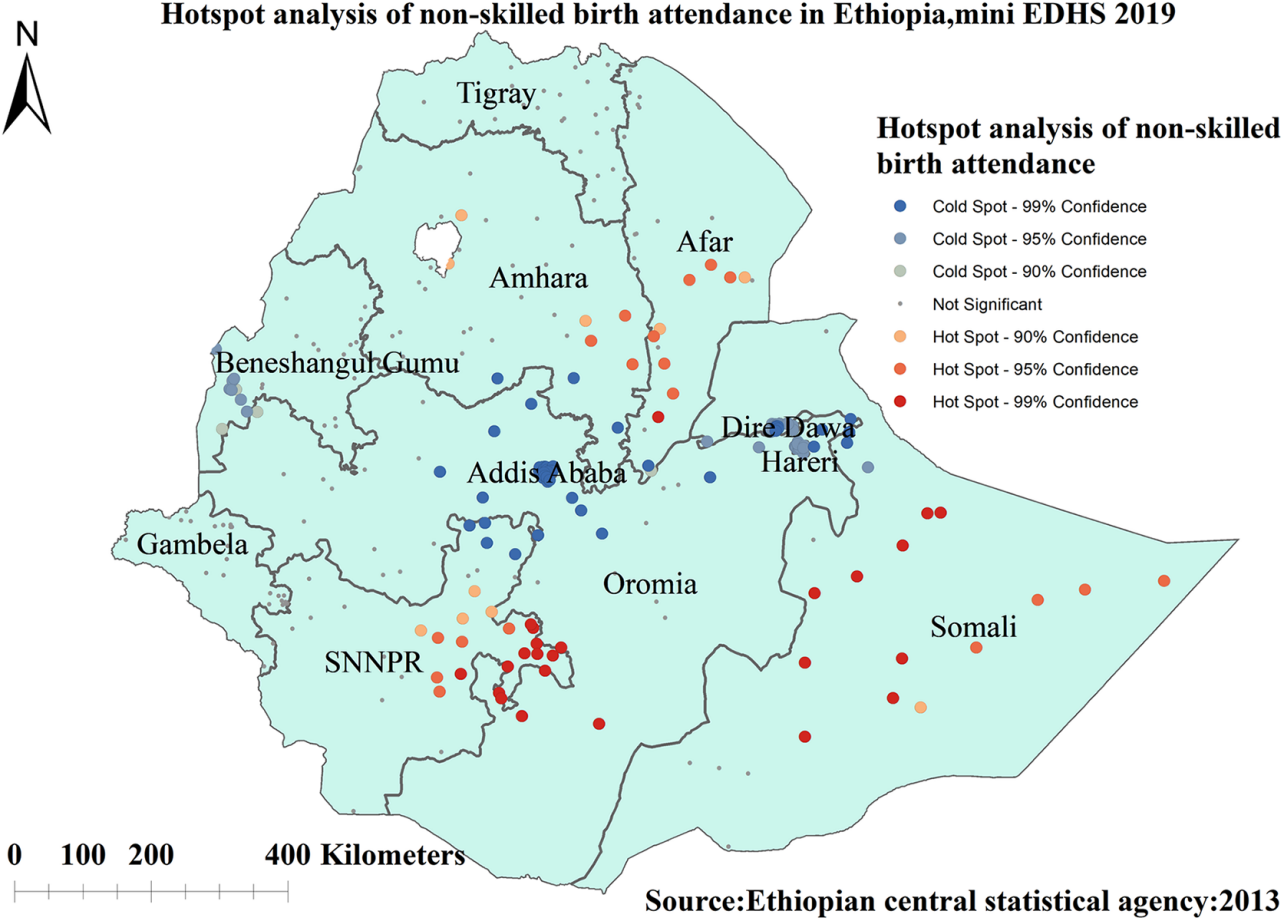
Hot spot analysis of non-skilled birth attendance among reproductive-age women in Ethiopia, 2019 mini EDHS.

The red color indicates regions with significant hotspot areas (areas with high rates of unskilled birth attendance), which were found in Somalia, SNNP, Afar, and southern parts of the Amhara regions. The blue color indicates areas/regions with significantly lower rates of unskilled birth attendance (cold spot areas), which were found in Addis Ababa, Dire Dawa, Harari, and western Benishangul Gumuz regions (Figure 4).

##### Kriging interpolation analysis

**Figure 5:**
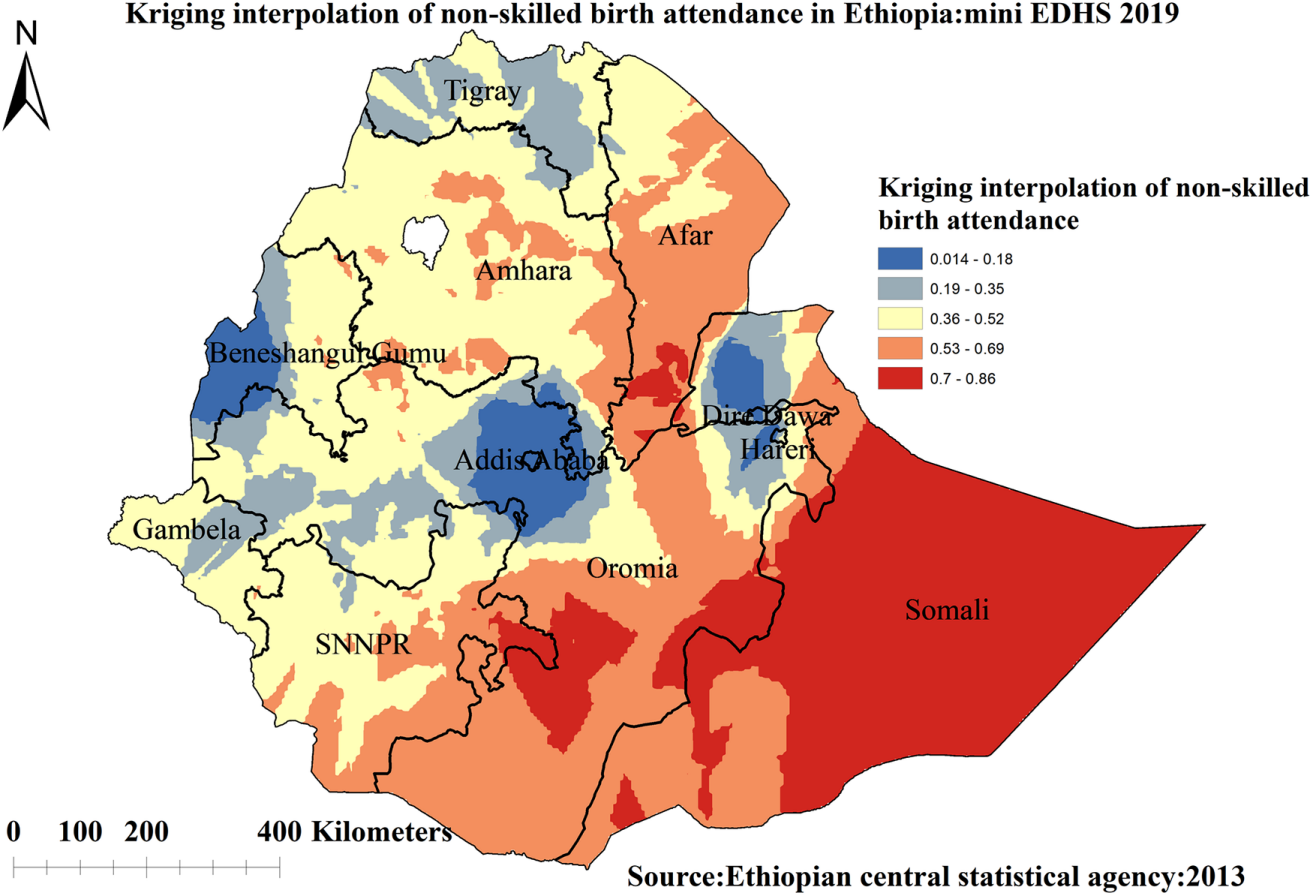
Kriging interpolation of non-skilled birth attendance among reproductive-age women in Ethiopia, 2019 mini EDHS.

Figure 5 indicated kriging interpolation methods of predicting the skilled birth attendance among reproductive-age women in Ethiopia. The predicted highest prevalence of unskilled birth attendance was detected in Somalia, Oromia, and some parts of the Afar regions of Ethiopia. In contrast, areas with the lowest prevalence of unskilled birth attendance were detected in Addis Ababa, Dire Dawa, Harari, and western Benishangul Gumuz regions of Ethiopia (Figure 5).

##### Spatial scan statistical analysis

**Figure 6:**
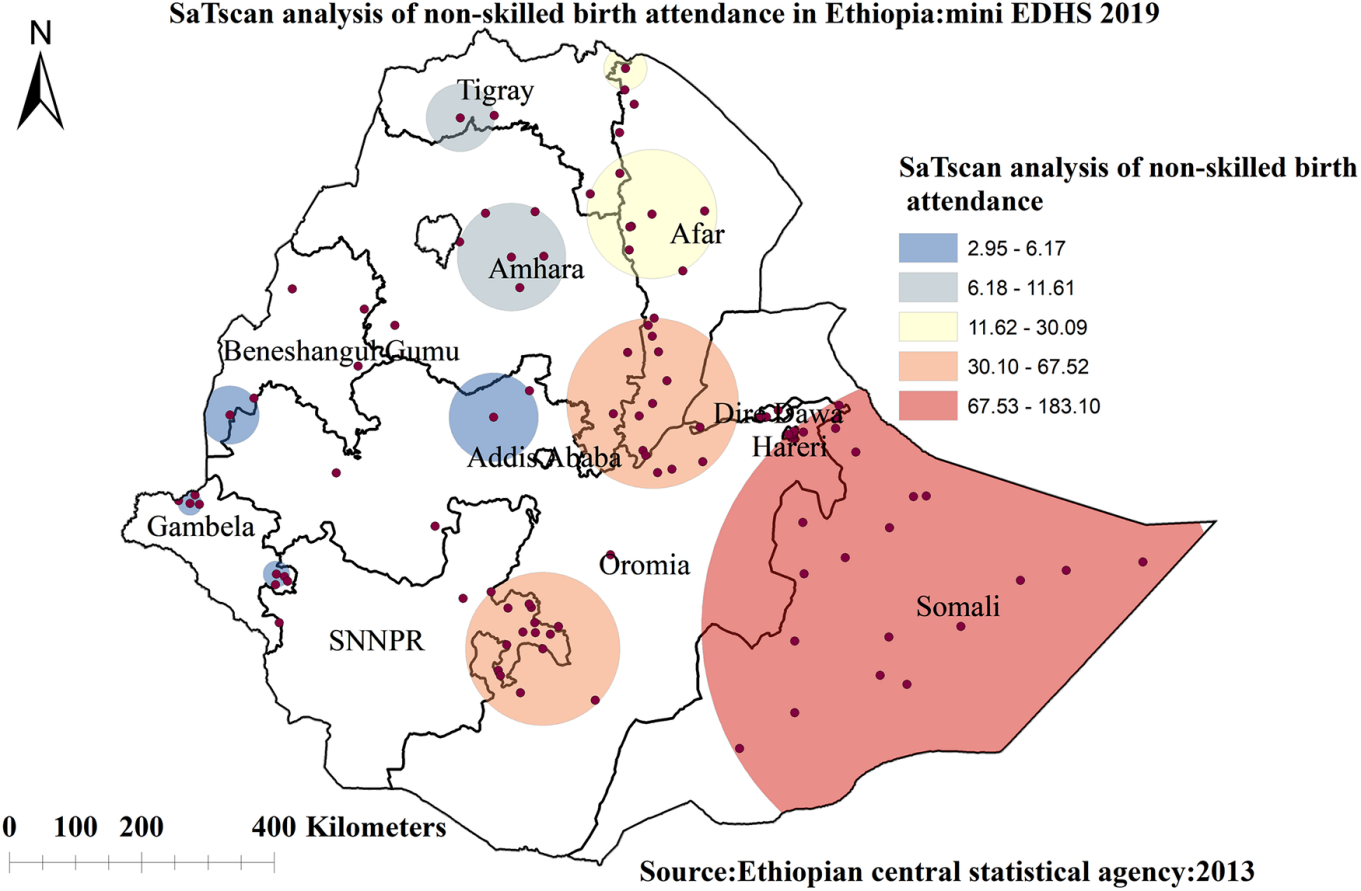
Spatial scan statistics analysis of non-skilled birth attendance among reproductive-age women in Ethiopia, 2019 mini EDHS.

A spatial scan statistical analysis identified a total of 71 significant clusters, of which 27 were most likely (primary) clusters, and 44 were secondary clusters. The primary clusters were located in Somalia and some parts of Oromia region centered at 6.639662 N, 44.465855 E with a 390.28 km radius, a Relative Risk (RR) of 1.88 and Log-Likelihood Ratio (LLR) of 183.09, at *p* < 0.001. It showed that women inside the spatial window had a 1.88 times higher likelihood of using skilled birth attendance than women outside the spatial window (Figure 6).

##### The fixed effect analysis result

In the multivariable mixed-effect logistic regression model; age, educational ANC Visit, having radio and television, parity, household size, wealth index, residence, community level poverty, and community level of women’s education were significantly associated with skilled birth attendance.

Women aged 35–49 were 49% more likely to use skilled birth attendance (AOR = 1.49, 95% CI; 1.11–2.00) as compared to women age group 15–24. The odds of skilled birth attendance among women who were married were increased by 54% (AOR = 1.54, 95% CI; 1.09–2.17) as compared to unmarried women. The odds of using skilled birth attendance among women who had primary education were increased by 84% (AOR = 1.84, 95% CI; 1.52–2.22) as compared to those women with no formal education. Similarly, women who had secondary education had 2.87 (AOR = 2.87, 95% CI; 2.00–4.11) times higher odds of skilled birth attendance than women with no formal education.

The odds of using skilled birth attendance among women with rich were increased by 83% (AOR = 1.83, 95% CI; 1.43–2.33) as compared to poor wealth status. The odds of skilled birth attendance among women who had television were 2.15 (AOR = 2.15, 95% CI = 1.45, 3.18) times higher as compared to women who had no television. The odds of using skilled birth attendance among women who had a radio were 22% (AOR = 1.22, 95% CI = 1.00, 1.49) higher as compared to women who had no radio.

Women who had ANC visits were 4.31 times (AOR = 4.31, 95% CI; 3.38–5.50) more likely to use skilled birth attendance than those who had no ANC visit. Multiparous women are 70% (AOR = 0.30, 95% CI; 0.23–0.40) lower odds of utilizing skilled birth attendance than primiparious women. Women having a household size of greater than six have 20% (AOR = 0.80, 95% CI = 0.66, 0.96) lower odds of having skilled birth attendance than women having a household size of 1–5.

Women living in high-community women's education had 77% (AOR = 1.77, 95% CI = 1.10,2.87) higher odds of using skilled birth attendance than women living in low-level community women's education. Women living in high community-level poverty were 64% (AOR = 0.36, 95% CI = 0.21, 0.60) lower odds of utilizing skilled birth attendance than women living in low-level community poverty. Women living in rural areas were 59% (AOR = 0.41, 95% CI; 0.21–0.80) less likely to use skilled birth attendance, compared to their counterparts in urban areas (Table 3).

**Table 3:** Multilevel multivariable analysis of factors associated with skilled birth attendance among reproductive-age women, 2019 mini EDHS.

Variables	Categories	Null model	Model I	Model II	Model III
			AOR [95% CI]	AOR [95% CI]	AOR [95% CI]
Age of women	15–24		1.00		1.00
	25–34		1.10 [0.87, 1.39]		1.05 [0.83, 1.33]
	35–49		1.63 [1.21, 2.18]		1.49 [1.11, 2.00]*
Marital status	Married		1.41 [1.00, 1.99]		1.54 [1.09, 2.17]*
	Not married		1.00		1.00
Women education status	No education		1.00		1.00
	Primary		1.96 [1.62, 2.37]		1.84 [1.52,2.22]***
	Secondary and higher		3.17 [2.22, 4.54]		2.87 [2.00, 4.11]***
Have television	No		1.00		1.00
	Yes		3.03 [2.07, 4.44]		2.15 [1.45, 3.18]*
Have radio	No		1.00		1.00
	Yes		1.20 [0.98, 1.47]		1.22 [1.00, 1.49]*
ANC visit	No		1.00		1.00
	Yes		4.36 [3.42, 5.56]		4.31 [3.38, 5.50]***
Parity	Primi		1.00		1.00
	Multi		0.29 [0.22, 0.39]		0.30 [0.23,0.40]***
Household size	<6		1.00		1.00
	≥6		0.77 [0.64, 0.93]		0.80 [0.66, 0.96]*
Wealth index	Poor		1.00		1.00
	Middle		1.30 [1.05, 1.62]		1.15 [0.92, 1.43]
	Rich		2.32 [1.82, 2.95]		1.83 [1.43, 2.33]***
Community level variables					
Residence	Rural			0.22 [0.11, 0.44]	0.41 [0.21, 0.80]**
	Urban			1.00	1.00
Community level poverty	Low			1.00	1.00
	High			0.24 [0.14, 0.41]	0.36 [0.21, 0.60]***
Community level of women's education	Low			1.00	1.00
	High			3.23 [1.95, 5.37]	1.77 [1.10, 2.87]*
Region	Small peripheral			1.00	1.00
	Large central			1.56 [0.88, 2.77]	1.23 [0.71, 2.12]
	Metropolitans			3.42 [1.36, 8.56]	1.71 [0.67, 4.32]

AOR, adjusted odds ratio; CI, confidence interval.

* *P*-value < 0.05.

** *P*-value < 0.01.

*** *P*-value < 0.001.

## Discussion

Only half of reproductive-age women in Ethiopia attend their delivery with skilled birth attendants. The finding is similar to previous studies done in Nigeria (56) and higher than in a previous study done in Ethiopia (57), and Kenya (58), and lower than a study done in Bangladesh (59), and Cameron (36). The possible reason for the variation might be that many interventions were implemented at different locations and times, potentially increasing access to a health facility and skilled birth delivery services. The Ethiopian health sector transformation plan of 2016–2020 set a nationwide skilled birth attendance delivery target of 90% of deliveries, which has not been reached yet as per the 2019 EMDHS (60). The possible reason for these discrepancies might be due to the high proportion of women of reproductive age in Ethiopia faced impediments to healthcare access (61).

The spatial analysis revealed that the spatial distribution of unskilled birth attendance varied across the country. The regions with high rates of unskilled birth attendance were found in the Somalia, SNNP, Afar, and southern parts of the Amhara regions. The possible reason for the large proportion of unskilled birth attendance in these regions could be due to barriers to healthcare access and limited access to mass media, resulting in a lower utilization of skilled birth attendance (62, 63). A low rate of unskilled birth attendance was observed in Addis Ababa, Diredawa, Harari, and western Benishangul Gumuz regions. The difference could be due to women living in metropolis regions might have a better socioeconomic status and better access to health services, allowing them to use of skilled birth attendance (40). The statistically significant primary clusters with a high prevalence of unskilled birth attendance Somalia and some parts of Oromia regions of Ethiopia. The finding is supported by previous studies done in Ethiopia (40).

Women aged 35–49 were more likely to use skilled birth attendance as compared to women aged 15–24. The finding is similar to a study done in Nigeria and Malawi (64), Nepal (24), and East Africa (32). This could be because older women have more autonomy and may have enough money which leads them to have an access to skilled delivery services (64). Furthermore, the risks of childbirth associated with advanced age may initiate older women to use skilled birth attendance (64).

Married women were more likely to utilize skilled birth attendance than unmarried women. The finding is similar to a study done in Nigeria and Malawi (64). One possible explanation is that married women might have more financial resources and support, allowing them to pay the fees for skilled maternal health care (25).

Women with primary and secondary education were more likely to use skilled birth attendance during delivery than women with no formal education. Also living in a community with a high level of women's education are more likely to utilize skilled birth attendance. The finding is similar to a study done in Ghana (65, 66), and Kenya (8). Furthermore, by promoting health awareness, education may influence women's healthcare-seeking behavior (67).

The study's findings also revealed that women in the rich wealth index were more likely to use skilled birth attendance than women in the poor wealth index. The finding is similar to a study done in Kenya (8), Ghana (65), Bangladesh (28), Cameroon (36), and a systematic review done in African countries (68). In many low- and middle-income countries, the cost of delivery may be a significant barrier to the use of skilled birth attendants (67). Additional costs for women seeking delivery care include transportation costs as well as opportunity costs such as travel time, waiting time, and lost productive activities (69, 70). Studies also indicated that the lack of cost to travel to health facilities is the main barrier to using skilled birth attendance in Ethiopia, although the majority of maternal health services in Ethiopia are provided free of charge (71).

Women having large households are less likely to use skilled delivery care than women's having fewer household sizes (72). The possible justification could be a lack of resources and time constraints due to family care and household responsibilities among women having large household sizes.

Women who had television and radio had higher utilization of skilled birth attendance than women who did not have television or radio. The finding is consistent with a study done in Sierra Leone, Niger, and Mali (35), East Africa (32), Bangladesh (28), and Cameroon (36). Furthermore, a study conducted in Guinea found that women who watched television at least once a week were more likely to use skilled birth attendance (6). Studies also indicated that the use of mass media had a positive influence on the utilization of health services (73). One possible explanation is that information shared through the media raises community awareness and fosters a positive attitude toward healthcare utilization, which encourages women to use skilled birth attendance.

Our findings show that women who received antenatal care are more likely to receive skilled birth attendance than women who did not receive antenatal care. The result is consistent with studies done in Ethiopia (22), Kenya (8), Uganda (31), East Africa (32), Zambia (33), Bangladesh (34), Sierra Leone, Niger, and Mali (35), and India (74). The ANC visit is a critical pillar for safe motherhood since it provides access to the continuum of maternity care, skilled delivery care, and a variety of essential services that promote the mother's and newborn's health (75). One essential part of ANC is sharing information and counseling women about birth preparation, as well as encouraging the value of skilled birth attendance.

The findings from this study indicated that as parity increases the likelihood of using skilled birth attendance at birth decreases. The finding is similar to a study done in Ethiopia (16, 30), East Africa (32), and Uganda (31). The possible explanation is that multiparous women perceive delivery as a usual process and choose unskilled support during delivery that will have the confidence to give birth at home (8, 69). Furthermore, a lack of prior negative experience with skilled birth attendant-assisted delivery among the primiparious may lead them to have more skilled birth attendance than multiparous women (76).

Also, we found lower utilization of skilled birth attendance among women in rural areas as compared to women living in urban areas. The result is consistent with studies done in Ethiopia (29, 30), South Sudan (27), Bangladesh (28), and Cameroon (36). A possible justification could be a lack of good road infrastructure and transportation facilities in rural communities, which could hinder the utilization of skilled birth attendance.

The main strength of the study was that it used nationally representative data with large sample size and a suitable statistical approach to accommodate the data's hierarchical nature. However, this study had limitations in that the cross-sectional nature of the data makes it impossible to infer causality between the independent and dependent variables. Since this survey relies on respondents’ self-report, there may be the possibility of recall bias because respondents were questioned to remember events from the past. Furthermore, because it was a mini report, the EMDHS data did not include information about some predictor variables of skilled birth attendance.

## Conclusion

Half of the women in Ethiopia did not utilize skilled birth attendance. Significant spatial clustering of unskilled birth attendance was observed in Ethiopia. Age, marital status, educational status, ANC Visit, having television and radio, parity, household size, wealth index, residence, community level poverty, and community level of women's education were significant predictors of skilled birth attendance. Skilled birth attendance was significantly and disproportionately concentrated in rich households (Pro-rich distribution). The regions of Somalia, SNNP, Afar, and southern Amhara were identified as having a high prevalence of using unskilled birth attendance. Public health interventions should target those women at high risk of using unskilled birth attendance and hotspot areas to improve use of skilled birth attendant uptake and improve maternal health. Policymakers should concentrate on enhancing health system coordination with multisectoral agents to improve skilled attendance at delivery.

## Data Availability

Publicly available datasets were analyzed in this study. This data can be found here: www.dhsprogram.com.
